# A phase I, randomized study of combined IL-2 and therapeutic immunisation with antiretroviral therapy

**DOI:** 10.1186/1476-8518-5-6

**Published:** 2007-04-11

**Authors:** Gareth AD Hardy, Nesrina Imami, Mark R Nelson, Ann K Sullivan, Ron Moss, Marlén MI Aasa-Chapman, Brian Gazzard, Frances M Gotch

**Affiliations:** 1 Department of Immunology, Imperial College London, Chelsea and Westminster Hospital, 369 Fulham Road, London, SW10 9NH, UK; 2 Department of HIV/GU Medicine, Imperial College London, Chelsea and Westminster Hospital, 369 Fulham Road, London, SW10 9NH, UK; 3 Immune Response Corporation, Carlsbad, CA, USA; 4 Wohl Virion Centre, Windeyer Institute of Medical Sciences, UCL, London, W1T 4JF, UK

## Abstract

**Background:**

Fully functional HIV-1-specific CD8 and CD4 effector T-cell responses are vital to the containment of viral activity and disease progression. These responses are lacking in HIV-1-infected patients with progressive disease. We attempted to augment fully functional HIV-1-specific CD8 and CD4 effector T-cell responses in patients with advanced chronic HIV-1 infection.

**Design:**

Chronically infected patients with low CD4 counts T-cell counts who commenced antiretroviral therapy (ART) were subsequently treated with combined interleukin-2 and therapeutic vaccination.

**Methods:**

Thirty six anti-retroviral naive patients were recruited and initiated on combination ART for 17 weeks before randomization to: A) ongoing ART alone; B) ART with IL-2 twice daily for 5 days every four weeks starting at week 17 for 3 cycles; C) ART with IL-2 as in group B and Remune HIV-1 vaccine administered once every 3 months, starting at week 17; and D) ART with Remune vaccine as in group C. Patients were studied for 65 weeks following commencement of ART, with an additional prior 6 week lead-in observation period. CD4 and CD8 T-cell counts, evaluations of HIV-1 RNA levels and proliferative responses to recall and HIV-1 antigens were complemented with assessment of IL-4-secretion alongside quantification of anti-HIV-1 CD8 T-cell responses and neutralizing antibody titres.

**Results:**

Neither IL-2 nor Remune™ vaccination induced sustained HIV-1-specific T-cell responses. However, we report an inverse relationship between HIV-1-specific proliferative responses and IL-4 production which continuously increased in patients receiving immunotherapy, but not patients receiving ART alone.

**Conclusion:**

Induction of HIV-1-specific cell-mediated responses is a major challenge in chronically HIV-1-infected patients even when combining immunisation with IL-2 therapy. An antigen-specific IL-4-associated suppressive response may play a role in attenuating HIV-specific responses.

## Background

Immune recovery subsequent to antiretroviral therapy (ART) often appears to be partial and does not comprise the HIV-1-specific CD4 or CD8 T-cell proliferative and IL-2-producing responses that are associated with protection from disease progression [1-5]. These potentially protective HIV-1-specific T-cell responses [6-9], become dysfunctional and exhausted with progressing disease. A number of approaches attempt modulation of cell-mediated responses, including therapeutic immunisation [2,10-12]. Remune™ is a whole, gp120-depleted, inactivated, HIV-1 immunogen in incomplete Freund's adjuvant (IFA) prepared from the recombinant primary isolate HZ-321 [13] (clade A envelope, clade G gag). Clinical trials of intramuscular (I/M) Remune™ including one phase III [14], have failed to demonstrate increases in disease-free survival time despite Remune's™ induction of HIV-1-specific CD4 T-cell responses [15]. Sub-group analysis failed to demonstrate any consistent effects on viral loads or CD4 counts [16]. Despite this, Remune™ may delay disease progression and reduce development of antiretroviral resistance [17].

Sub-cutaneous (S/C) interleukin (IL)-2, administered with ART, increases CD4 T-cell numbers [18-21] and recall antigen-specific CD4 lymphocyte proliferation [22,23]. However timing may be crucially important to the induction of cell-mediated responses [24]. We have previously shown that IL-2 administration subsequent to immunization was associated with boosted responses to the antigen in question, suggesting a therapeutic role for IL-2 in enhancing proliferative T-cell responses in HIV-1 infection [2,25].

We investigated the ability of Remune™ and IL-2, combined and separately, to induce HIV-1-specific CD4 and CD8 T-cell responses in chronically HIV-1-infected patients on ART in an observational, open-label, randomized, pilot study. We also assessed antigen-specific IL-4 release as this cytokine plays a role in balance and/or suppression of cell-mediated responses [26,27], We report here evaluation of specific T-cell proliferation, antigen-specific IL-4 release, CD8 T-cell IFN-γ responses and neutralizing antibody titres, in order to comprehensively describe the specific immune response relevant to control of viral replication.

## Methods

### Patients and Study Design

In this observational, phase I, pilot study conducted at Chelsea and Westminster Hospital, London, 36 antiretroviral-naive patients were initiated on ART at week 0, which was continued for the duration of the study. ART comprised 2 nucleoside analogues and one protease inhibitor or non-nucleoside reverse transcriptase inhibitor. At week 17 patients were randomized to receive immunotherapy with IL-2 and/or therapeutic immunisation with a gp120-depleted whole inactivated HIV-1 immunogen. Sufficient Remune™ was donated for use in 20 patients by Immune Response Corporation (IRC), Carlsbad, CA, USA. Patients were randomized at week 17 only if their viral load was <50 copies ml/plasma and CD4 T-cell count was ≥ 300 cells/μl blood at week 16. Treatment groups for randomization were as follows: A) ART alone (n = 9); B) ART plus IL-2 (Proleukin™) (n = 11); C) ART plus IL-2 and Remune™ (n = 7); and D) ART plus Remune™ (n = 9). IL-2 (5 × 10^6^U) was administered S/C, twice daily, for 5 days at weeks 17, 21 and 25. 100 μg Remune™ was administered I/M at weeks 17, 29, 41 and 53. Laboratory analysis was conducted at weeks- -6, -3 and 0 before ART, and weeks 1, 2, 4, 8, 16, 17, 21, 25, 29, 41, 53 and at study completion at week 65. The primary outcome was induction of positive changes in lymphocyte proliferative responses to HIV-1 antigens. In addition to the main study time points, a further sub-study of viral loads and lymphocyte subset numbers was conducted in a sub-set of patients (n = 15) receiving IL-2 in groups B and C on the 5^th ^day of each IL-2 cycle, i.e. at weeks 18, 22 and 26. This sub-study was initiated after the main study had begun and included all patients receiving IL-2 in groups B and C from the date of its inception. Appropriate regulatory approval was granted by Riverside Ethics Committee and patients gave written informed consent.

### Separation of PBMCs

PBMC were separated from whole blood by density gradient centrifugation and cultured in RPMI-1640 with NaCO_3 _(Sigma, Poole, UK) and 100 IU/ml penicillin, 100 μg/ml streptomycin and 2 mM L-glutamine supplemented with 10% human AB plasma (all Sigma).

### Viral Load and Lymphocyte Subsets

Plasma viral load was measured using the Bayer HIV-1 RNA 3.0 assay (bDNA) (lower limit 50 copies/ml) (Bayer Diagnostics, Newbury, UK). Whole blood lymphocytes were counted with monoclonal antibodies to: CD3, CD4, CD8, and CD45 (Tetra One, Beckman Coulter, High Wycombe, UK) on an Epics XL-MCL flow cytometer (Beckman Coulter).

### Recall and HIV-1 antigens

Recombinant HIV-1 and recall antigens (Medical Research Council Centralised Facility for AIDS Reagents, National Institute for Biological Standards and Controls, Potters Bar, UK) were used as described previously [2], at a final concentration of 10 μg/ml. Remune™ and its native p24 (IRC, Ca) were used at 3 μg/ml [28].

### Lymphocyte Proliferative Assays

Proliferation assays and supernatant collection for IL-4 assessment were conducted as previously described [2]. Stimulation indices (SI) for triplicates (standard error <15%) were calculated as antigen-stimulated β-particle counts per minute (cpm)/no antigen cpm. A positive response was regarded as a SI >5.

### Measurement of Cytokine Production

IL-4 bioassays were carried out as previously described [2] using IL-4 (CT.h4S) dependent cell lines. Briefly, IL-4 was measured in culture supernatants of HIV-1 antigen stimulated proliferation assays. Proliferation of the IL-4-dependent cell line CT.h4S was measured by incorporation of tritiated thymidine. Results are presented as IL-4 driven cpm.

### Delayed hypersensitivity (DTH) tests to Remune™

*In vivo *delayed-type hypersensitivity (DTH) skin tests to Remune™ antigen were performed to assess HIV-1-specific cell-mediated immune responses as described elsewhere [29] in all patients at weeks 17, 29 and 53.

### HLA-typing

HLA haplotypes of patients were assessed by PCR-SSP [30].

### IFN-γ ELIspot assays

IFN-γ ELIspots were conducted as described previously [31]. Briefly, 2.5 × 10^5 ^PBMC from individual patients who had been tissue typed were cultured with or without 10 μg/ml of appropriate HLA-restricted peptides or PHA (positive control) in 96-well anti-IFN-γ (Mabtech, Stockholm, Sweden) coated PVDF-backed plates (Millipore, Watford, UK). After overnight incubation IFN-γ spot-forming cells (SFC) were detected according to the manufacturer's instructions (Mabtech).

### Neutralisation assays

Immunoglobulin (Ig)G was purified from patient and HIV-1 negative plasma using the MAbTrap kit (Amersham Biosciences, Little Chalfont, UK), and quantified by Protein Assay (Bio-Rad, Munich, Germany). Two-fold serial dilutions of purified IgG, from 1 mg/ml, were incubated with 100 focus forming units (FFU) of HIV-1_SF162 _for 1 hr at 37°C and then plated onto NP2/CD4^+^/CCR5^+ ^cells. Infection was detected after 48 hrs by p24-immunostaining, as detailed before [32]. The percentage reduction of infection was calculated: 100 × (1-(mean FFU with patient IgG)/(mean FFU with seronegative IgG at 1 mg/ml)).

### Statistical Analysis

Changes in lymphocyte subsets and viral load between week 0 and 16, weeks 0 and 65 and weeks 16 and 65, were assessed using the Wilcoxon Signed Rank test. Proliferation and IL-4 data were assessed with repeated measures analysis of variance using MIXED procedure in SAS statistical software. For log-transformed antigen-specific proliferative stimulation indices, between and within subject weeks 0 to 17, weeks 17 to 65 and weeks 0 to 65, adjusted separate slopes were estimated for each study arm with 95% confidence interval. Differences in overall viral load from week 18–26, between groups B and C were assessed using the Pearson Chi-squared test.

## Results

### Patient demographics

Fifty two anti-retroviral naïve patients were screened for this study which was carried out at the Chelsea and Westminster Hospital, London over a six year period. Of the screened patients 16 dropped out prior to randomisation at week 17. All the remaining 36 patients who were randomised completed the study. The mean age of patients at study entry was 38.75 years. There was one female patient (2.78%) and 100% of patients were of white European ethnicity.

### Viral loads and CD4 T-cell counts

**Figure 1:**
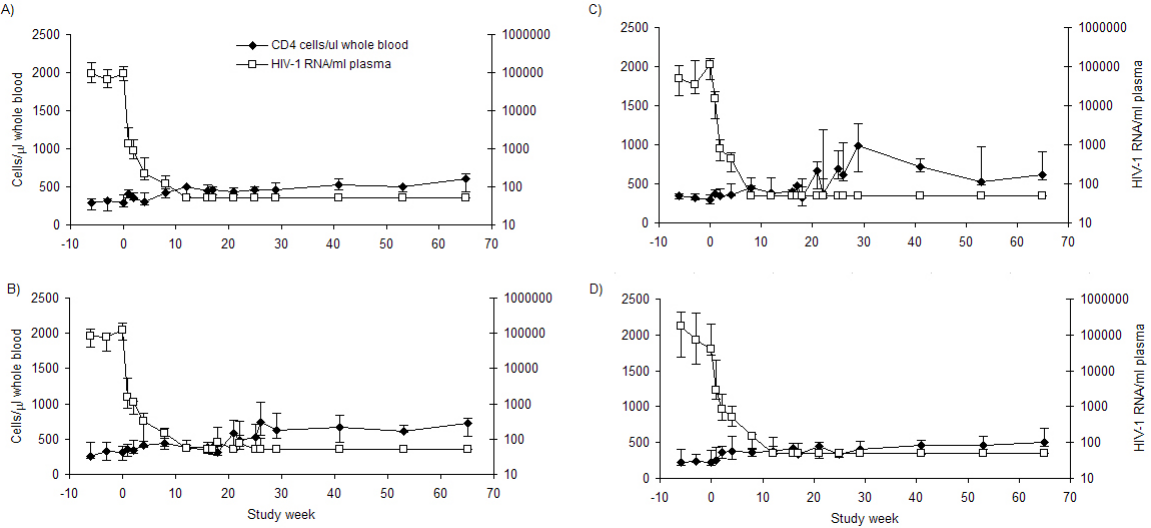
Median absolute CD4 T-cell counts per μl of whole blood (closed diamonds) and median HIV-1 RNA viral load per ml of plasma (open squares) with 1st and 3rd interquartile ranges over the entire study period for group A (A), group B (B), group C (C), and group D (D). A subset of patients were assessed for viral load and CD4 T-cell counts at day 5 of each IL-2 cycle in groups B and C. Results are shown for all patients. Elevations in viral loads occurred more often in viral loads of patients receiving ART and IL-2 without Remune™ (group B), than in patients receiving ART and IL-2 with Remune™ (group C).

Median viral loads and CD4 T-cell counts for each group are depicted in Figure 1. The median week 0 viral load for all patients was 88,699 copies/ml plasma (range 50–779,254). The median absolute CD4 T-cell count was 294 cells/μl whole blood (range 76–551). Nineteen of 36 patients (53%) had CD4 T-cell counts below 300 cells/μl blood at pre-ART time points.

By week 17 the median viral load in all groups was less than 50 copies. All patients, except 5 (one in Group A, three in Group B, one in group D), maintained viral suppression at the main study time points from week 17 onwards while receiving ongoing ART. One patient in Group C (patient 11) elected to discontinue ART at week 47 causing subsequent rebound in viraemia. At week 17 the median CD4 T-cell count in each complete group was as follows: Group A, 456 cells/μl (range 261–673) (Figure 1A); Group B, 377 cells/μl (range 81–742) (Figure 1B); Group C, 468 cells/μl (range 306–680) (Figure 1C); Group D, 337 cells/μl (range 289–702) (Figure 1D). IL-2-induced increases in absolute CD4 T-cell counts are also apparent in groups B and C. By week 65 these values were 602 cells/μl (range 300–918) for Group A, 731 cells/μl (range 253–1025) for Group B, 619 cells/μl (range 358–1404) for Group C and 505 cells/μl (range 345–768) for Group D.

### Lymphocyte proliferation to HIV-1 antigens

**Figure 2:**
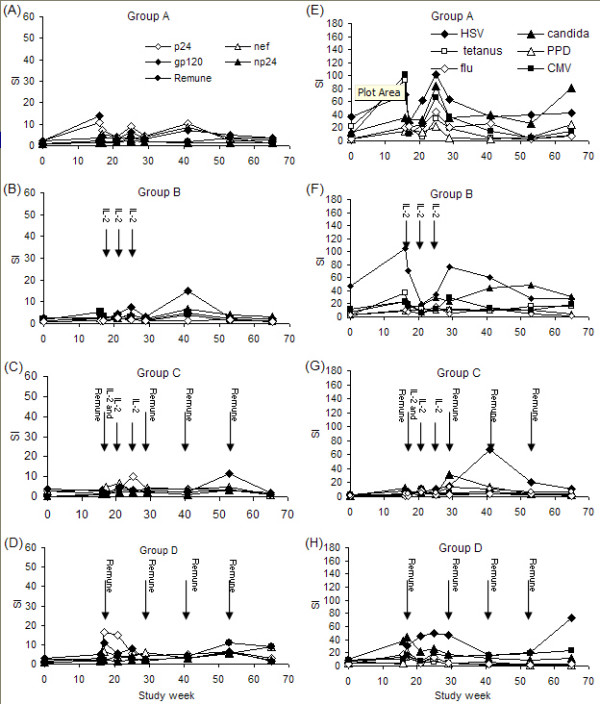
Mean HIV-1-specific lymphocyte proliferative responses (left-hand panel) with the times of IL-2 and Remune™ administration, showing responses for group A (A), group B (B), group C (C) and group D (D) and mean recall-specific lymphocyte proliferative responses (right-hand panel) with the times of IL-2 and Remune™ administration, for group A (E), group B (F), group C (G) and group D (H). The scale of HIV-1-specific responses is displayed as half the magnitude of that for recall responses.

HIV-1-specific proliferative responses increased transiently for many patients. Regression analysis of these responses revealed no significant changes in group A (Fig. 2A). In group-B from week 0 to 65 a significant increase in the np24 response (*p *= 0.005) was observed (Fig. 2B). In group C no increase in HIV-1-specific proliferative responses were seen (Fig. 2C). Of note was an outstanding response to Remune™ with an SI of 95 at week 65 for patient 11 in this group (data not shown) coincident with rebound viraemia (152,536 copies/ml) following self-imposed ART discontinuation. In group D a positive regression was seen in responses to Nef (*p *= 0.004) and whole Remune™ antigen (*p *= 0.005) from week 0 to 65. (Fig. 2D). The only response which significantly increased between week 17 and 65 in any group was the p24 response in group D (*p *= 0.039). Patient 8 in this group demonstrated a very large response to Remune™ (SI = 261) and np24 (SI = 144) at week 29 following breakthrough of resistant virus at week 25 (6,315 copies/ml) (data not shown). No other substantial increases in responses occurred between week 17 and 65 for any group or at any time point between the groups.

### *In vivo *delayed type hypersensitivity reactions to Remune™

Induration size of hypersensitivity reactions to Remune™ did not become positive (>9 mm diameter) in any patient at any of the time points tested (weeks 17, 29 and 53).

### Lymphocyte proliferation to recall antigens

Baseline to week 16 T-cell responses have been described in detail previously [1]. Regression analysis revealed significant increases in recall responses in all groups between week 0 and 65 (Fig. 2E–H), predominantly for the *persistent *recall antigens: For group A (Figure 2E) responses to CMV and Candida significantly increased (*p *= 0.027 and *p *= 0.006 respectively); for group B (Figure 2F) HSV responses significantly increased (*p *= 0.007), CMV responses increased *p *= 0.002 and Candida responses increased (*p *= 0.0006); in group C (Figure 2G) CMV and Candida responses increased (*p *= 0.002 and *p *= 0.007 respectively); in group D (Figure 2H) HSV and CMV responses significantly increased (*p *= 0.002 and *p *= 0.001). Responses to *transient *antigens were less apparent. In group A PPD and tetanus responses showed significant positive regressions over the 65 weeks (*p *= 0.018 and *p *= 0.018 respectively); in group B only the PPD response regression curve was significant (*p *= 0.044); and in group C only the tetanus response was significant (*p *= 0.0003). There were no significant differences in recall responses between treatment groups.

### HIV-1-specific IL-4 production

**Figure 3:**
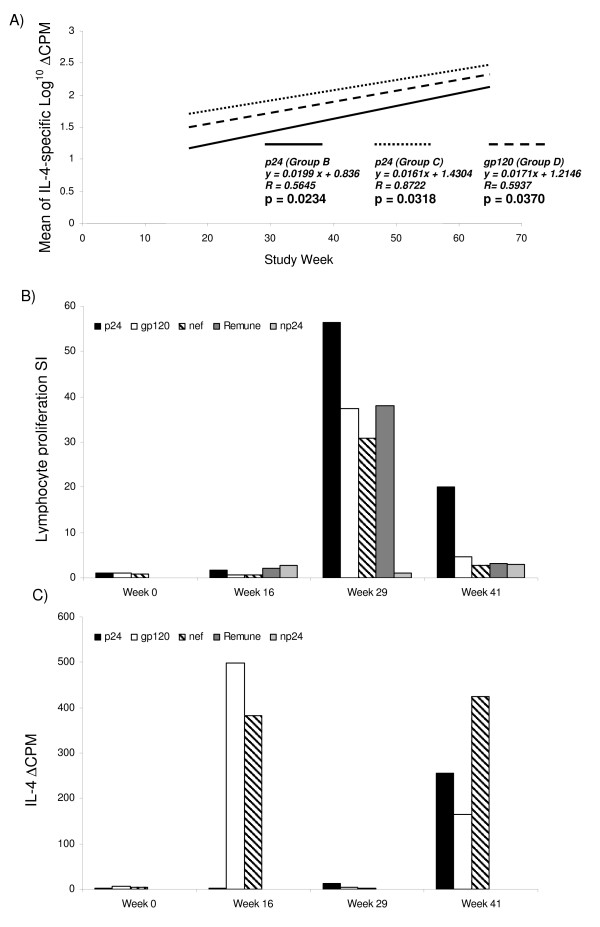
HIV-1-specific IL-4 production and lymphocyte proliferative responses. Significant increases in CT.h4S delta cpm in response to HIV-1-specific IL-4 production were seen over the week 17–65 period (A) for groups B (ART + IL-2), C (ART + IL-2 + Remune™) and D (ART + Remune™), but not for group A (ART alone) (data not shown). Patient 11 is shown as an example of an inverse relationship between lymphocyte proliferative stimulation index (SI) (B) and IL-4 production as delta cpm (C) which was evident for 17 of 28 patients who were assessed for IL-4 production.

Antigen-specific IL-4 production may be associated with suppression of proliferative responses and dampening of inflammatory immune responses. Therefore we measured IL-4 production in proliferation assay culture supernatants in response stimulation with each HIV-1 antigen, as previously described [1]. Significant increases in HIV-1-specific IL-4 production from weeks 17 to 65 were observed in response to p24 in group-B (*p *= 0.023), and group C (*p *= 0.032) and in response to gp120 in group D (*p *= 0.037) (Fig. 3A). HIV-1-specific IL-4 production did not significantly increase in group A (data not shown).

We observed an apparent relationship between IL-4 production and loss/lack of proliferation in 17 of 28 patients tested, distributed evenly between groups, evident from week 0 to 16 and week 17 to 65. Patient 11 (group C) is shown as an example, where HIV-1-specific proliferative responses to p24, gp120, nef and Remune™ are high at week 29 (Fig. 3B) with absence of IL-4 (Fig. 3C), while at week 41 IL-4 secretion increases as proliferation declines.

### CD8 T-cell IFN-γ responses to HLA class I restricted peptides

Where possible we investigated CD8 T-cell IFN-γ production in response to characterised HLA class I restricted peptides (listed in Table 1), by ELISpot assay using cryo-preserved PBMCs. As reported previously [15] Remune™ did not induce increases in CD8 T-cell responses. Although fluctuations occurred in CD8 T-cell IFN-γ responses these were not sustained and could not be attributed to ART or immunotherapy. Summary data is presented from week 0, following ART alone at week 16 and at week 65 in Table 2. Assays in which responses were not observed to the postive control, PHA, were considered to have failed, as denoted by "F". Of note is the observation that no patient in groups B, C or D demonstrated an increase in responses between week 16 and week 65. Patient 11 is an exception, although this patient had high baseline responses and by week 65 had discontinued ART causing considerable rebound viraemia, concomitant with strong HIV-1-specific lymphocyte proliferative responses as detailed above.

**Table 1:** HLA class I restricted peptides used for IFN-γ ELISpot assays

Peptide	Protein	Sequence	HLA-restriction
Gag			

g1	p17 (71–79)	GSEELRSLY	A1
g2	p17 (77–85)	SLYNTVATL	A*0201
g3	p17 (20–28)	RLRPGGKKK	A3
g4	p24 (217–227)	ACQGVGGPGHK	A11
g5	p17 (84–92)	TLYCVHQRI	A11
g6	p17 (28–36)	KYKLKHIVW	A24
g7	p24 (35–43)	EVIPMFSAL	A26
g8	p24 (16–23)	SPRTLNAW	B7
g9	p17 (74–82)	ELRSLYNTV	B8
g10	p24 (197–205)	DCKTILKAL	B8
g11	p17 (93–101)	EIKDTKEAL	B8
g12	p17 (24–31)	GGKKKKYKL	B8
g13	p24 (131–140)	KRWIILGLNK	B27
g14	p17 (36–44)	WASRELERF	B35^A^
g15	p17 (124–132)	NSSKVSQNY	B35^A^
g16	p24 (122–130)	PPIPVGDIY	B35^A^
g17	p24 (174–184)	AEQASQDVKNW	B44
g18	p24 (83–92)	VHPVHAGPIA	B55
g19	p24 (108–117)	TSTLQEQIGW	B57^B^
g20	p24 (176–184)	QASQEVKNW	B57^B^
g21	p24 (15–23)	ISPRTLNAW	B57^B^
g22	p24 (32–40)	FSPEVIPMF	B57^B^
g23	p17 (92–101)	IEIKDTKEAL	B61

Pol			

p1	RT (309–317)	ILKEPVHGV	A*0201
p2	RT (179–187)	VIYQYMDDL	A2
p3	RT (485–493)	ALQDSGLEV	A2
p4	RT (341–350)	IYQEPFKLNK	A11
p5	RT (158–166)	AIFQSSMTK	A11
p6	IN (179–188)	AVFIHNFKRK	A11
p7	RT (448–457)	RETKLGKAGY	A29
p8	RT (392–401)	PIQKETWETW	A32
p9	RT (18–26)	GPKVKQWPL	B8
p10	RT (175–183)	HPDIVIYQY	B35^A^
p11	RT (156–164)	SPAIFQSSM	B35^A^
p12	RT (432–441)	EPIVGAETFY	B35^A^
p13	RT (203–212)	EELRQHLLRW	B44
p14	RT (397–406)	TWETWWTEYW	B44

Nef			

n1	nef (180–189)	VLEWRFDSRL	A2
n2	nef (73–82)	QVPLRPMTYK	A11
n3	nef (75–82)	PLRPMTYK	A11
n4	nef (84–92)	AVDLSHFLK	A11
n5	nef (128–137)	TPGPGVRYPL	B7
n6	nef (90–97)	FLKEKGGL	B8
n7	nef (13–20)	WPTVRERM	B8
n8	nef (135–143)	YPLTFGWCY	B18/B49
n10	nef (186–193)	DSRLAFHH	B35^A^
n11	nef (74–84)	VPLRPMTY	B35^A^
n12	nef (116–125)	HTQGYFPDWQ	B57^B^
n13	nef (92–100)	KEKGGLEGL	B61

Peptides were identified from the NIH HIV Molecular Immunology Database website [50]. References for each peptide are also available at this website. RT (reverse transcriptase antigen), IN (integrase antigen). ^A^HLA B35 is associated with rapid progression in HIV-1 infection. ^B^HLA B57 is associated with slow/non progression in HIV-1 infection

**Table 2:** Patient HLA types and ELISpot results, showing the sum of delta (background subtracted) IFN-γ spot forming cells (SFC) at weeks 0, 16 and 65 for each patient for whom ELISpot analysis was possible.

Patient	HLA type (peptides used)	Sum of Δ IFN-γ SFC/10^6 ^PBMC								
		
		Week 0			Week 16			Week 65		
Group A		Gag	Pol	Nef	Gag	Pol	Nef	Gag	Pol	Nef

1	A1 (g1) A2 (g2, p1, p2, p3, n1) B7 (g8, n2) B18 (n8)	20	20	260	0	0	150	20	10	680
16	A2, (g2, p1, p2, p3, n1) B44 (g17, p13, p14)	50	90	110	940	850	320	740	1200	20
36	A2, (g2, p1, p2, p3) A23, (g13) B27, (n9) B49 (n8)	F	F	F	70	60	30	230	280	370
52	A11, (g4, g5, p5, n2) A9, (none identified) B35^A^, (g14, p10) B61 (g23, p15, n13)	670	470	295	910	405	1280	370	215	165
54	A2, (g2, p2, n1) A11, (g5, p5) B27, (n9) B35^A ^(g14, p10)	80	190	30	F	F	F	290	240	160

Group B		Gag	Pol	Nef	Gag	Pol	Nef	Gag	Pol	Nef

7	A2, (g2, p2, p3, n1) A24, (g6) B55, (g18) B57^B ^(g19, g20, 21, g22)	500	30	0	1310	120	20	1340	190	0
35	A3, (g3) A32, (p8) B7 (g8, n5)	50	110	75	130	70	25	185	135	115
57	A1, (g1) B8 (g9, g10, g11, g12, p9, n6, n7)	490	50	130	190	30	20	450	70	120

Group C		Gag	Pol	Nef	Gag	Pol	Nef	Gag	Pol	Nef

4	A1, (g1) A26, (g7) B27, (n9) B57^B^(g19, g20, g21, g22, n12)	220	nd	50	130	nd	40	520	nd	60
11	A1, (g1) A2, (g2, p1, p2, p3, n1) B35^A^, (g14, g15, g16, p10, p11, p12, n10, n11) B57^B^(g19, g20, g21, g22, n12)	3820	3140	2990	90	50	20	2820	2760	960
14	A1, (g1) A2, (g2, p1, p2, p3, n1) B57^B^,(g19, g20, g21, g22, n12) B62 (none identified)	F	F	F	1140	130	20	100	30	70
34	A3, (g3) A29, (p7) B35^A^, (g14, g15, p10, n10) B44 (g17, p13, p14)	320	100	0	F	F	F	520	80	10

Group D		Gag	Pol	Nef	Gag	Pol	Nef	Gag	Pol	Nef

13	A2, (g2, p1, p2, n1) A11, (g4, p4, n2) B8, (g9, p9, n6) B62 (none identified)	50	20	70	730	1890	1190	390	640	240
22	A1, (g1) A2, (g2, p2, n1) B7, (g8, n5) B35^A ^(g14, g15, p10, n11)	270	80	120	F	F	F	60	40	260

F denotes assay failure. IFN-γ (interferon-gamma), PBMC (peripheral blood mononuclear cells), SFC (spot forming cells). ^A^HLA B35 is associated with rapid progression in HIV-1 infection. ^B^HLA B57 is associated with slow/non progression in HIV-1 infection.

### Discordant viraemia between patients receiving IL-2 with or without Remune™

Secondary to the main study protocol, further viral load and lymphocyte subset analysis was conducted at additional time points – week 18, week 22 and week 26 – for a sub-group of 15 patients receiving IL-2 in groups B (n = 9) and C (n = 6) on the last day of each IL-2 cycle. Small, transient elevations in HIV-1 RNA load were apparent on 12 occasions for 7 of 9 (78%) patients in group-B, and 2 of 6 patients (33%) in group-C. All these viral load elevations had spontaneously resolved by the next viral load test 3 weeks later at weeks 21, 25 and 29. The difference in occurrence of these viral load elevations remains evident when comparing the median viral loads for all the patients in groups B and C as shown in Fig. 1B and 1C. The median viral load change (from 50 copies/ml) for the patients who had day 5 viral load measurements in group B were 23.5 copies/ml, 53 copies/ml and 0 copies/ml at the end of each IL-2 cycle. In comparison the median change in viral load for patients in group C who had day 5 viral load measurements was 0 at each time point. Change in viral load was not significantly different when comparing the sub-study patients in group B with those in group C, at any of the 3 individual IL-2 day 5 time points. However when taking all three day 5 time points together for each group, comparison between the two groups revealed a trend towards significance (*p *= 0.071, Pearson Chi-Squared).

### Neutralising antibody responses in discordant virologic responders

As Remune™ displays the transmembrane (gp41) envelope antigen with strong neutralization epitopes [33-35], it may prime neutralizing antibodies to new epitopes. We tested the ability of plasma IgG from patients in Group C who did not experience day 5 viral blips to neutralize HIV-1_SF162 _at weeks 0, 16 and 65. There was no effect of immunotherapy or ART on virus neutralization (data not shown).

## Discussion

In this pilot study of combined immunotherapy with the therapeutic HIV-1 vaccine Remune™ and subcutaneous IL-2 in the context of ART, neither IL-2 nor Remune™ immunisation confered any substantial benefits to the effect of ART with regard to HIV-1-specific CD4 or CD8 T-cell responses, or neutralising antibodies. For the majority of these chronically HIV-1-infected patients responses to HIV-1 antigens remained negligible and there was little difference between the four immunotherapy arms. Possible reasons for this could be: the low CD4 T-cell counts of these patients when ART was initiated resulting in protracted immunosuppression and/or clonal dysfunction; a need for longer duration of ART before immunotherapeutic intervention, thus allowing greater immune recovery; the permanent depletion of HIV-1-specific CD4 T-cells by direct HIV-1 infection; and/or the timing of IL-2 therapy with immunisation which may have important consequences for induction or suppression of induced responses.

Previous Remune™ studies which induced proliferative responses comprised patients with higher pre-ART mean CD4 T-cell counts of 617 cells/μl [36] and 700 cells/μl blood [15]. The mean in this study was 303 cells/μl. 53% of these patients had CD4 nadirs below 300 cells/μl. Immune reconstitution in patients who initiate ART with low CD4 T-cell counts is impaired compared to those who initiate ART with higher CD4 T-cell counts [37,38]. The ability to respond to immunisation is also dependant on higher CD4 T-cell count nadirs and earlier initiation of ART [39].

The fate of missing HIV-1-specific CD4 T-cell responses is unclear. HIV-1 preferentially infects and deletes many HIV-1-specific CD4 T-cells [7], while some remain detectable, expressing IFN-γ, though unable to proliferate or express IL-2 [40,41]. The significant increase in HIV-1-specific IL-4 production from weeks 17 to 65 in group B (p24), C (p24) and D (gp120) could indicate clonal dysfunction in these patients. Only group A (ART alone) had no increase in HIV-1-specific IL-4 production. Thus immunotherapy in these patients appears to be associated with increasing levels of HIV-1-specific IL-4 production. IL-4, an anti-inflammatory type-2 cytokine, has a suppressive effect on lymphocyte proliferation [26,42]. The observation in a number of patients that HIV-1-specific IL-4 increases when proliferative responses diminish may indicate a mechanism by which clonal proliferation remains suppressed, although this needs further investigation. We previously found a balanced IL-4/IL-2 phenotype in HIV-1-specific CD4 T-cell responses in patients who remain disease free [27]. While IL-4 expression by HIV-1-specific CD4 T-cells may be thought of as a pathological anti-proliferative effect, it must be considered that this phenotype could be protective, by dampening immune activation and quashing viral replication. This relationship requires further investigation.

Despite the lack of induced HIV-1-specific T-cell responses in these patients we report significant increases in recall antigen proliferative responses, particularly for *persistent *antigens. These increases were largely evident between week 0 and 65 with no differences between groups, suggesting immunotherapy was ineffective in this respect. While some significant increases in proliferative responses to HIV-1 antigens were apparent from week 0 to 65 (group B and D), the same cannot be said regarding the immunotherapy period between weeks 17 and 65. Nor were there any significant differences in responses between groups. These results suggest that any improvements were singularly attributable to duration of ART.

Of note is one patient in group B, vaccinated with tetanus 4 weeks before receiving IL-2. High tetanus-specific proliferation was enhanced and sustained by subsequent IL-2, as reported separately [25]. In contrast a second patient received tetanus vaccination after IL-2 and did not respond. These responses may be dependent on administration of IL-2 subsequent to antigen priming, during T-cell contraction. Animal models demonstrate IL-2 administration during T-cell contraction enhances and prolongs responses, unlike co-administration of IL-2 with antigen when the survival of responding T cells is abrogated [24]. Our protocol and others that also achieved limited induction of responses [43] utilized a co-administered model. Future trials will address such important issues.

We observed low-levels of transient self-limiting viraemia resulting from IL-2 therapy as previously reported [44]. Despite the lack of detectable cell-mediated responses induced by immunotherapy in these patients, the transient viral load blips we have reported appear to occur less in patients receiving IL-2 when administered with Remune™ in group C, with a trend towards significance (p = 0.071). Larger group sizes may have revealed a stronger relationship between Remune™ and protection from IL-2 induced transient viraemia, but this pilot study was not powered for such observations. This possible effect of Remune™ was not due to induction of neutralising antibodies, by the display of potentially sensitive epitopes on gp41 resulting from the removal of gp120 from Remune™ [45]. We have separately reported the induction of antibody responses against HLA-B62 and HLA-DR4 in some of these patients who received Remune™ immunisation [46], as Remune™ contains these antigens derived from the HUT-78 cell line in which it is grown. Group sizes are too small in this study to determine whether such responses may have played a role in potential protection from viral blips in group C.

## Conclusion

Combined Remune™ and IL-2 with ART in advanced HIV-1 infection conferred no immunological benefits to ART. Taking together the absence of induced HIV-1-specific lymphocyte proliferative responses, CD8 T-cell IFN-γ responses and *in vivo *DTH responses these results imply that induction of renewed HIV-1-specific cell-mediated responses by therapeutic immunisation, even when supplemented with IL-2, is extremely problematic in patients who initiate ART with lower CD4 T-cell counts. Although a recently reported clinical trial of the vCP1433 canary pox-based therapeutic vaccine elicited p24-specific responses which were significantly associated with time off therapy in a subsequent treatment discontinuation protocol, these responses also remained transient, diminishing at study end [47]. This underscores the difficulties in inducing protective immune responses by therapeutic immunisation of chronically infected patients. Furthermore immunological protection in chronically infected individuals may be best conferred by autologous virus, as opposed to vaccine derived antigens as found with the vCP1452 canary pox vaccine [48] to which T-cell responses may in fact be associated with more rapid viral rebound following treatment interruptions [49]. In our study we saw the greatest proliferative responses to HIV-1 antigens in patients who experienced virological rebounds, demonstrating that autologous virus induces greater responses, albeit transiently, than immunisation or IL-2 therapy.

In these chronically infected treated patients, we found that immunotherapy was associated with increasing HIV-1-specific IL-4 production, which appears to negatively impact proliferative responses. HIV-1-specific IL-4 production may result from a general dysfunction of HIV-1-specific CD4 T-cells with pathological implications for induction of HIV-1-specific responses. We suggest that these results underscore the importance of early initiation of ART. As Remune™ may have positive effects in less advanced patients [17], we suggest further investigations, with or without cytokine adjuvants, be conducted in patients for whom extensive immunological damage has been prevented with earlier initiation of ART.

## Abbreviations

ART (antiretroviral therapy), CMV (Cytomegalovirus), cpm (counts per minute), DNA (Deoxyribonucleic Acid), HIV-1 (Human Immunodeficiency Virus type-1), HLA (Human Leukocyte Antigen), HSV (Herpes Simplex Virus), IgG (Immunoglobulin G), IFN (Interferon), IL (Interleukin), I/M (Intramuscular), PBMC (Peripheral Blood Mononuclear Cells), PCR (Polymerase Chain Reaction), RNA (Ribonucleic Acid), S/C (subcutaneous), SI (Stimulation Index).

## Competing interests

The author(s) declare that they have no competing interests.

## Authors' contributions

All authors have read and approved the final version of this manuscript. GH, FG, NI and BG conceived the study and co-coordinated the design of the study. GH and BG participated in the application for government and ethical approval, and in co-ordination of the study; he conducted laboratory work and cellular immunological assays, conducted data analysis and Interpretation, and preparation and completion of the manuscript. NI had responsibility for overall management and coordination of the study, she conducted laboratory work and cellular immunological assays, coordinated data analysis and interpretation, and participated in preparation of the manuscript. FG and NI secured funding for the study. FG participated in data analysis and interpretation, and in preparation of the manuscript. RM provided Remune™ vaccine and Remune™ antigen and its native p24 antigen and participated in study design. MAA conducted virus neutralisation assays. BG participated in the design of the study, co-coordinated application for government and ethical approval, and coordinated patient management. AS undertook patient care and management and participated in study co-ordination, and data interpretation. MN participated in patient care and management and data interpretation.
